# Mobile Health–Based Thermometer for Monitoring Wound Healing After Endovascular Therapy in Patients With Chronic Foot Ulcer: Prospective Cohort Study

**DOI:** 10.2196/26468

**Published:** 2021-05-07

**Authors:** Donna Shu-Han Lin, Jen-Kuang Lee

**Affiliations:** 1 Division of Cardiology Department of Internal Medicine National Taiwan University Hospital Taipei Taiwan; 2 Cardiovascular Center National Taiwan University Hospital Taipei Taiwan; 3 Department of Laboratory Medicine National Taiwan University College of Medicine Taipei Taiwan; 4 Telehealth Center National Taiwan University Hospital Taipei Taiwan; 5 Department of Internal Medicine, College of Medicine National Taiwan University Taipei Taiwan

**Keywords:** temperature, peripheral artery disease, endovascular therapy, mHealth, app, foot, therapy, wound, thermometer, monitoring, ulcer, artery, prospective, cohort, healing

## Abstract

**Background:**

Foot temperature may increase after endovascular therapy, but the relationship between foot temperature and wound healing is unclear.

**Objective:**

This study was performed to evaluate the feasibility of a mobile health (mHealth)–based thermometer for foot temperature monitoring in patients with chronic foot ulcer before and after endovascular therapy and to determine the association between temperature change and wound healing time.

**Methods:**

This was a prospective cohort study. Patients who had a chronic foot ulcer (>3 months) and underwent endovascular therapy between July 2019 and December 2019 were included. The participants received standard medical care and endovascular therapy for revascularization. The mHealth-based thermometer, composed of 4 temperature-sensing chips, was put on the foot before and after endovascular therapy. Data from the chips were transferred to an associated mobile phone app via Bluetooth. Wound healing time was estimated using the Kaplan-Meier method, and the associations between baseline characteristics and clinical outcomes were evaluated using a Cox proportional hazard model.

**Results:**

A total of 163 patients with chronic foot ulcer who underwent endovascular therapy were enrolled and followed up until wound healing was complete or for 180 days. The mean foot temperature before endovascular therapy was 30.6 (SD 2.8 °C). Foot temperature increased significantly (mean 32.1 °C, SD 2.8 °C; *P*=.01) after the procedure. Wound healing time was significantly different in the Kaplan-Meier curves of the patient group with temperature changes ≥2 °C and the group with temperature changes ≤2 °C (log-rank *P*<.001). A foot temperature increase ≥2 °C after endovascular therapy was associated with increased wound healing in univariate analysis (hazard ratio [HR] 1.78, 95% CI 1.24-2.76, *P*=.02), and the association remained significant in multivariate analysis (HR 1.69, 95% CI 1.21-2.67, *P*=.03).

**Conclusions:**

The mHealth-based thermometer was feasible and useful for foot temperature monitoring, which may provide health care professionals with a new endpoint for endovascular therapy. Foot temperature increases ≥2 °C after endovascular therapy were associated with faster wound healing in patients with chronic foot ulcer. Further studies are needed, however, to confirm these findings.

## Introduction

Chronic foot ulcer is a disease associated with multiple conditions [1], such as old age, diabetes, smoking, and chronic kidney disease. It often occurs in patients with the most extreme cases of lower extremity arterial disease and inflammatory cardiac disease [2,3]. Patients presenting with minor or major tissue loss (Rutherford stages V and VI) are at high risk of future amputation [4]. Current guidelines recommend reperfusion with bypass surgery or endovascular therapy for patients with critical limb ischemia to minimize tissue loss [5]. Optimal endpoints in endovascular therapy are important to determine wound recovery in order to prevent future amputation.

Although the guidelines [5,6] suggest the use of an in-line revascularization strategy for endovascular therapy, various procedural techniques and outcomes have been advocated. Iida et al [7] reported that angiosome-guided endovascular therapy improved wound healing rates compared to the wound healing rates of non–angiosome-guided procedures, emphasizing the concept of anatomy. Kawarada et al [8] found that skin perfusion pressure, a physiology-based parameter, was useful in determining when to end endovascular therapy. Moreover, transcutaneous oxygen pressure, near-infrared spectroscopy, wound blushing phenomenon, and vascular flow reserve have all been studied as endovascular therapy outcome measures [9-14]. It has been reported that periwound foot temperature is associated with wound status [15]. Foot temperature rises immediately after endovascular therapy, but the relationship between foot temperature and wound healing in endovascular therapy has never been studied.

The use of traditional thermometers in measuring foot temperature is limited by their poor sensitivity. With the newest technology, however, a nanochip thermometer can be used to measure temperature in various places—solid objects, air, skin, etc—easily, rapidly, and accurately. Connecting nanotechnology to mobile health (mHealth) is currently a trend for health monitoring and diagnosis to improve health care and optimize individualized medicine. With mHealth, patients can operate, record, and upload medical information to cyberspace through mobile apps independently, that is, without clinic visits or doctor consultations.

We aimed to investigate the feasibility of using an mHealth-based thermometer to monitor foot temperature in patients with chronic foot ulcer and lower extremity arterial disease before and after endovascular therapy. In addition, we examined whether increases in foot temperature were associated with wound healing times in these patients.

## Methods

### Study Design

This study was a prospective cohort study performed at a single tertiary medical center in Taipei, Taiwan. The study was conducted with the approval of the institutional review board of National Taiwan University Hospital (201906078RIPD) and in accordance with the Declaration of Helsinki [16]. All participants provided written informed consent both for the procedure and for inclusion in the study cohort.

### Patient Selection

Patients with chronic foot ulcers were consecutively enrolled from the outpatient clinic or during admission from July 2019 to December 2019 if they were adults scheduled to undergo endovascular therapy of the iliac artery, superficial femoral artery, popliteal artery, anterior tibial artery, peroneal artery, and posterior tibial artery. Demographic, clinical, and laboratory data were obtained prospectively, with a corroborative retrospective monthly review performed for all patients who underwent endovascular therapy through quality-control reports of the catheterization laboratory. The exclusion criteria for the study were patients with an active infection, with Rutherford stage VI ischemia, who did not ultimately receive endovascular therapy, and without recorded pre– or post–endovascular therapy foot temperature. Patients had follow-up visits at the outpatient department in our hospital at least every 3 months for wound healing assessment.

### mHealth-Based Thermometer

**Figure 1:**
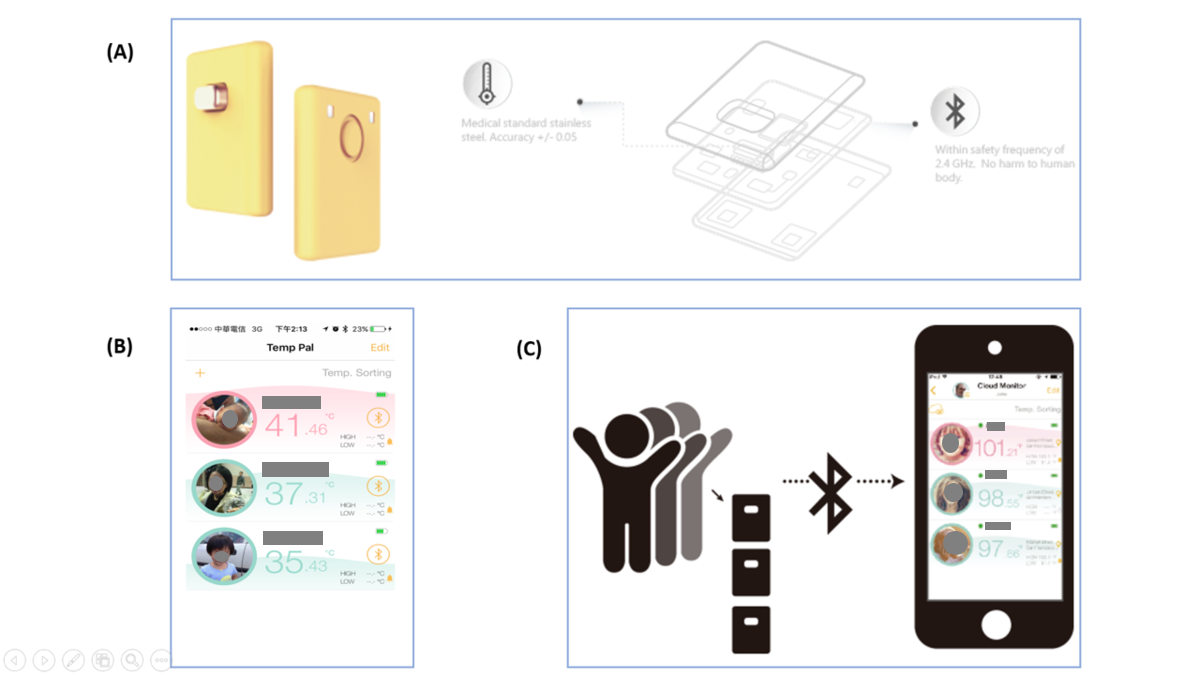
(A) Temp Pal body-temperature monitoring nanochip-based thermometer and app, which can (B) monitor multiple devices simultaneously and (C) transfer data via Bluetooth.

Temp Pal (iWEECARE Co Ltd; Figure 1) is a wireless continuous body temperature monitoring device that allows simultaneous recordings at multiple spots. We used 4 devices paired with one iOS mobile device and the Temp Pal app via Bluetooth (Figure 1) to capture continuous temperature data, which were uploaded to the cloud and exported. With an auto-calibration algorithm, the accuracy and precision of the Temp Pal is within ±0.05 °C.

.

### Outcome Measures

The primary endpoint was complete wound healing, which was defined as complete epithelialization of the wound determined with visual assessment performed by doctors. Patients were followed up for up to 180 days after endovascular therapy or until wound healing was complete, whichever occurred first. We used time-to-event analysis for all outcomes. Secondary outcomes were change in foot temperature at the instep of the foot and at the sole after endovascular therapy.

### Statistical Analysis

Data were analyzed using SPSS software (version 22; IBM Corp). Statistical significance was considered to be indicated by 2-sided *P*<.05. Nonnormally distributed continuous data are reported as median and IQR. The chi-square test and Fisher exact test were used to compare differences in discrete and categorical variables (sex, diabetes, hypertension), respectively. The *t* test or the Wilcoxon rank-sum test was to compare continuous variables (foot temperature) pre– and post–endovascular therapy. The correlation between instep and sole temperature was examined using Pearson correlation.

The association between demographic, clinical, and laboratory characteristics with wound healing 180 days after endovascular therapy was determined with univariate logistic regression analysis. Variables achieving statistical significance (*P*<.10) in the univariate analysis were included in multivariate logistic regression analysis. Collinearity testing was done to avoid interdependence between the model variables. Hazard ratios (HRs) with 95% CIs are reported.

The threshold of foot temperature increase associated with wound healing was identified in the multivariate analysis. Time-to-wound healing was compared between patients whose foot temperature increase was greater than and those whose foot temperature increase was less than this threshold using the log-rank test or Gehan-Breslow-Wilcoxon Test. The Kaplan–Meier technique was used to estimate the cumulative incidence of complete wound healing in 180 days for each of the 2 groups.

## Results

### Patient Demographics and Clinical Features

**Figure 2:**
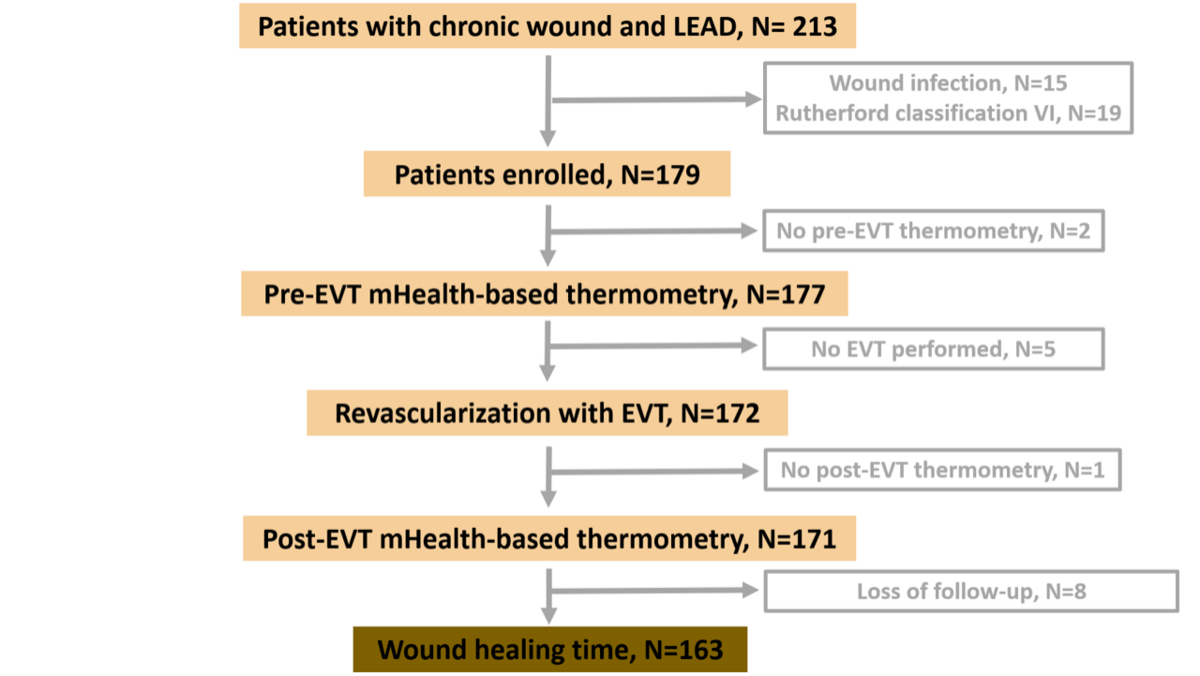
Participant flowchart. LEAD: lower extremity arterial disease; EVT: endovascular therapy.

Between July 2019 and December 2019, a total of 213 patients who had a chronic foot ulcer for longer than 3 months signed informed consent forms and were screened for enrollment (Figure 2). Of these, 15 patients were excluded for clinical signs of wound infection, and 19 patients were excluded due to Rutherford stage VI ischemia. Among the remaining 179 patients, 2 patients did not undergo pre–endovascular therapy foot temperature measurement, 5 patients did not undergo endovascular therapy, and 1 patient did not undergo post–endovascular therapy temperature measurement. Eight other patients were lost to follow-up during the study period; therefore, 163 patients were included in the analysis.

The mean follow-up was 133 days (SD 55). The mean age of the patients was 72.6 years (SD 13.1), and 104 patients (63.8%) were male. The mean BMI was 24.3 kg/m^2^ (SD 4.5). There were 50 (30.6%) patients who were wheelchair-bound or bedridden. Notable comorbidities included coronary artery disease (125/163, 76.7%), diabetes (123/163, 75.4%), hypertension (128/163, 78.5%), dyslipidemia (112/163, 68.7%), and dialysis dependence (37/163, 22.6%). The majority of patients were taking antiplatelet medication (140/163, 85.8%), and 138 (84.6%) patients were taking medication for diabetes (hemoglobin A_1c_ level: median 7.8%, IQR 2.7%). Baseline demographic and clinical characteristics are shown in Table 1.

**Table 1:** Baseline patient characteristics.

Characteristic		Value (n*=*163)
Age, mean (SD)		72.6 (13.1)
BMI^a^, mean (SD)		24.3 (4.5)
**Sex, n (%)**		
	Male	104 (63.8)
	Female	59 (36.2)
**Ambulatory, n (%)**		
	Yes	113 (69.3)
	No	50 (30.7)
**Risk factors, n (%)^b^**		
	Current smoking	44 (26.9)
	Diabetes	123 (75.4)
	Hypertension	128 (78.5)
	Dyslipidemia	112 (68.7)
	Chronic kidney disease	89 (54.6)
	Dialysis	37 (22.6)
	Atrial fibrillation	34 (21.0)
	Coronary artery disease	125 (76.7)
	Congestive heart failure	34 (20.8)
	Stroke	20 (12.2)
Left ventricular ejection fraction (%), median (IQR)		55.2 (16.0)
Hemoglobin A_1C_ (%), median (IQR)		7.8 (2.7)
**Medications, n (%)^c^**		
	Antiplatelet	140 (85.8)
	Anticoagulation	34 (20.8)
	Beta-blocker	63 (38.6)
	Calcium channel blockers	86 (52.7)
	Angiotensin converting enzyme inhibitors/angiotensin receptor blockers	94 (57.6)
	Oral antidiabetic drugs	138 (84.6)
	Insulin	34 (20.8)

^a^BMI: body mass index.

^b^Percentages do not add to 100 since patients may have multiple comorbidities.

^c^Percentages do not add to 100 since patients may take multiple medications.

### Foot Temperature Before and After Endovascular Therapy

Instep temperature was correlated with sole temperature both before (*r=*0.93) and after (*r=*0.92) endovascular therapy. Before endovascular therapy, instep and sole temperatures were mean 30.8 °C (SD 3.0 °C) and mean 30.4 °C (SD 2.8 °C), respectively, and the foot temperature before endovascular therapy was mean 30.6 °C (SD 2.8 °C). After endovascular therapy, instep and sole temperatures were mean 32.6 °C (SD 2.9 °C) and mean 32.0 °C (SD 2.8 °C), respectively, and the foot temperature after endovascular therapy was mean 32.1 °C (SD 2.8 °C). The differences in instep (*P*=.005), sole (*P*=.009), and foot (*P*=.01) temperatures before and after endovascular therapy were statistically significant.

### Prognosis of Wound Healing Time With Foot Temperature Change After Endovascular Therapy

**Figure 3:**
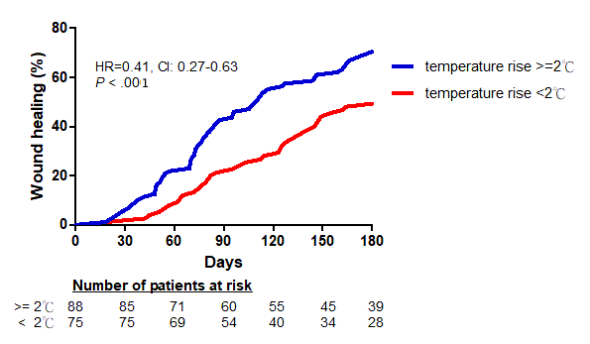
Wound healing time Kaplan-Meier curves (log-rank test *P*=.01; for trend *P*<.007). HR: hazard ratio.

Patients were categorized into those whose foot temperature increased by ≥2 °C (n=75) and those whose foot temperature increased by *<*2 °C (n=88) after endovascular therapy, in whom wound healing was achieved in 61 and 37 patients, respectively, over the course of the follow-up period. Wound healing occurred in a significantly lower percentage of patients whose foot temperature increased by *<*2 °C (HR 0.41; 95% CI 0.27-0.63; *P*<.001) than in those whose foot temperature increased by ≥2 °C (Figure 3).

### Predictors of Wound Healing Time After Endovascular Therapy

Univariate analysis revealed that a nonambulatory status (HR 1.68, 95% CI 1.13-1.92, *P*=.04) and foot temperature increase >2 °C (HR 1.78, 95% CI 1.24-2.76, *P*=.02) were significantly predictive of wound healing within 180 days. Dialysis dependence was significantly negatively associated with wound healing within 180 days (HR 0.87, 95% CI 0.61-0.96, *P*=.03). In the multivariate Cox analysis, these associations remained significant (ambulatory status: HR 1.71, 95% CI, 1.18-1.91, *P*=.03; foot temperature increase ≥2 °C: HR 1.69; 95% CI, 1.21-2.67, *P*=.03; dialysis dependence: HR 0.81, 95% CI 0.63-0.92, *P*=.02).

**Table 2:** Univariate and multivariate analyses: predictors of wound healing at 180 days after endovascular therapy.

Variable	Univariate analysis		Multivariate analysis	
	HR^a^ (95% CI)	*P* value	HR (95% CI)	*P* value
Age *<*80 years	1.54 (0.91-1.78)	.07	1.41 (0.82-1.86)	.15
Female	1.26 (0.74-1.56)	.45	1.15 (0.71-1.56)	.59
BMI^b^ *>*18.5 kg/m^2^	1.65 (0.85-1.88)	.32	1.46 (0.81-1.96)	.43
Not ambulatory	1.68 (1.13-1.92)	.04	1.71 (1.18-1.91)	.03
Diabetes	0.76 (0.45-1.46)	.22	0.87 (0.65-1.78)	.35
Hypertension	1.13 (0.61-1.52)	.56	1.19 (0.68-1.46)	.51
Dyslipidemia	1.27 (0.54-2.67)	.66	1.25 (0.61-2.28)	.48
Dialysis	0.87 (0.61-0.96)	.03	0.81 (0.63-0.92)	.02
Δ Foot temperature ≥2 °C	1.78 (1.24-2.76)	.02	1.69 (1.21-2.67)	.03

^a^HR: hazard ratio.

^b^BMI: body mass index.

## Discussion

### Principal Findings

We investigated mHealth-based monitoring of foot temperature change after endovascular therapy in patients with chronic foot ulcer. We demonstrated that a temperature increase >2 °C after endovascular therapy was strongly associated with faster healing time.

The endpoints currently used for endovascular therapy in clinical practice for critical limb ischemia vary. Wound bed temperature is crucial to wound healing—collagen deposition and the activities of late-phase inflammatory cells and fibroblasts are impeded when the temperature of the wound bed is lower than the core body temperature, leading to delayed healing [17]. It has been demonstrated in vitro that neutrophil, fibroblast, and epithelial cell activities decrease below a critical temperature of 33 °C [18]. Although the mean foot temperature after endovascular therapy in our cohort was less than this cut-off, our results showed that an increase in foot temperature is also an effective and reliable endpoint and may be a good surrogate endpoint for revascularization in critical limb ischemia.

However, increased local temperature is also a classic sign of wound infection. We, therefore, excluded patients with signs of local wound infection in the foot. Previous studies have shown that even mildly elevated temperatures may suggest occult infection that may lead to amputation [19]. Fierheller et al [20] found that integrating quantitative skin temperature measurements into routine wound assessment is a prompt and dependable means of quantifying the heat associated with skin infection and monitoring wound status. At-home self-monitoring with daily foot thermometry may thus be an effective adjunctive tool for preventing complications in individuals at high risk of lower-extremity ulceration and amputation. Combined with telehealth care, mHealth-based thermometry is a promising technology for treating chronic foot ulcer during the acute stage after endovascular therapy and the chronic stage of wound infection control.

mHealth is a popular trend, at present, that facilitates precise and individualized health care for various diseases. With the help of mobile devices, patients can easily record and transmit health-related parameters to a large database or to clinicians for consultation. Boodoo et al [21] demonstrated that patients were interested in mHealth for preventing and monitoring diabetic foot ulcers, even though some participants were not frequent users of mobile technology. However, not all mHealth-based monitoring options are appropriate for chronic foot ulcer treatment. Wearable sensors embedded in dressings or bandaging, for real-time monitoring of variables such as pH and temperature in diabetic foot ulcers, and telehealth platforms with medical-grade cameras are available on the market; however, they are often cumbersome and expensive. van Netten et al [22] suggested that images capture using mobile phones should not be used alone because they demonstrate low validity and reliability for the remote assessment of diabetic foot ulcers. Numerous mHealth studies of diabetes-related complications are now in progress, including studies on diabetes retinopathy [23] and chronic foot ulcer [21], which are associated with worsened cardiovascular prognoses. mHealth-based treatment continues to show potential in improving patient outcomes.

Adherence to frequent outpatient clinic visits may be difficult for patients with chronic foot ulcer who are older adults, bedridden or wheelchair-bound, and dependent on others for performing activities of daily living. Without intensive medical treatment, chronic foot ulcers are not likely to heal, which may result in further infection or amputation. The use of mHealth-based thermometry with telehealth can provide personalized evaluation and treatment and easy access to medical services: Lazo-Porras et al [24] reported that the implementation of foot thermometry through SMS prevented diabetic foot ulcers [24]. Recent reviews have found that SMS reminders were useful in several clinical scenarios, such as in enhancing adherence to antiretroviral [25], tuberculosis medications [26], and smoking cessation [27]. The system used in our study, a mobile phone–based thermometer with Bluetooth and app to transmit data automatically to the cloud, and would be easier for clinicians to use than SMS. The idea of combining mHealth and telehealth in caring for vulnerable patients is particularly attractive, and further clinical trials are needed to assess its efficacy.

Patients with diabetes and chronic foot ulcers exhibit poor prognoses in terms of cardiovascular outcomes; telehealth care can be used to provide comprehensive medical treatment beyond wound treatment alone. A previous study [28] has shown that telehealth care exerts positive effects in terms of disease control, including for hypertension, dyslipidemia, and diabetes, all of which are important predisposing factors for chronic foot ulcer, but chronic foot ulcer is strongly associated with concomitant cardiovascular disease, and therefore, its presence poses an increased risk of cardiovascular events [29]. In a previous study [30], we found that patients with cardiovascular disease who were receiving telehealth care had improved clinical outcomes.

### Limitations

This study has several limitations. First, it was a single center cohort study with a limited number of patients; however, to the best of our knowledge, the study cohort is the largest cohort to date addressing the impact of an mHealth intervention on patients receiving endovascular therapy. Second, the existence of numerous confounding factors in our cohort study may have influenced the results. We used a multivariate Cox analysis model to diminish the possible confounding effects of other clinical factors, but conducting randomized trials would be advisable to verify our findings. Third, we measured the temperature before and after patients underwent endovascular therapy, with the assistance of study nurses; however, it would be challenging for patients in the target population (wheelchair-bound, bedridden) to use this modern device for daily at-home temperature self-monitoring. Development of a user-friendly interface, ideally with single-button activation, is necessary in the next generation mHealth-based device to meet the needs of these patients. Lastly, wound healing was defined by complete epithelialization of the wound as observed by the eye. Different wound locations and wound sizes on the foot may have influenced the rate of wound healing.

### Conclusion

We demonstrated that increases in foot temperature by >2 °C after endovascular therapy, recorded with mHealth-based thermometers, were associated with better wound healing in patients with chronic foot ulcer. In the future, the mHealth-based thermometers may be combined with telehealth medicine for comprehensive care of diabetes patients with chronic foot ulcer. Multicenter studies, with longer follow-up, are needed to validate these results and provide guidelines for clinical practice.

## Abbreviations

HR: hazard ratio
mHealth: mobile health
SMS: short message service
