# Correction: Mobile Health–Based Thermometer for Monitoring Wound Healing After Endovascular Therapy in Patients With Chronic Foot Ulcer: Prospective Cohort Study

**DOI:** 10.2196/39749

**Published:** 2022-06-01

**Authors:** Donna Shu-Han Lin, Jen-Kuang Lee

**Affiliations:** 1 Division of Cardiology Department of Internal Medicine National Taiwan University Hospital Taipei Taiwan; 2 Cardiovascular Center National Taiwan University Hospital Taipei Taiwan; 3 Department of Laboratory Medicine National Taiwan University College of Medicine Taipei Taiwan; 4 Telehealth Center National Taiwan University Hospital Taipei Taiwan; 5 Department of Internal Medicine, College of Medicine National Taiwan University Taipei Taiwan

## Abstract

In the “Patient Selection” section in Methods, “Patients with chronic foot ulcers were consecutively enrolled from the outpatient clinic or during admission from June 2019 to December 2019” should instead be “Patients with chronic foot ulcers were consecutively enrolled from the outpatient clinic or during admission from July 2019 to December 2019”. Similarly, in the “Methods” section in the Abstract, “Patients who had a chronic foot ulcer (>3 months) and underwent endovascular therapy between June 2019 and December 2019 were included” should read “Patients who had a chronic foot ulcer (>3 months) and underwent endovascular therapy between July 2019 and December 2019 were included”. Lastly, in the “Patient Demographics and Clinical Features” section in Results, “Between June 2019 and December 2019” should instead be “Between July 2019 and December 2019”.

In “Mobile Health–Based Thermometer for Monitoring Wound Healing After Endovascular Therapy in Patients With Chronic Foot Ulcer: Prospective Cohort Study” (JMIR Mhealth Uhealth 2021;9(5):e26468) the authors noted three errors.

1. In the “Methods” section in the *Abstract*, “Patients who had a chronic foot ulcer (>3 months) and underwent endovascular therapy between June 2019 and December 2019 were included” has been corrected to “Patients who had a chronic foot ulcer (>3 months) and underwent endovascular therapy between July 2019 and December 2019 were included.”
2. In the “Patient Selection” section in *Methods*, “Patients with chronic foot ulcers were consecutively enrolled from the outpatient clinic or during admission from June 2019 to December 2019” has been corrected to “Patients with chronic foot ulcers were consecutively enrolled from the outpatient clinic or during admission from July 2019 to December 2019.”
3. In the “Patient Demographics and Clinical Features” section in *Results*, “Between June 2019 and December 2019” has been corrected to “Between July 2019 and December 2019.”

The correction will appear in the online version of the paper on the JMIR Publications website on June 1, 2022, together with the publication of this correction notice. Because this was made after submission to PubMed, PubMed Central, and other full-text repositories, the corrected article has also been resubmitted to those repositories.

